# Prognostic Value of the Lactate/Albumin Ratio for 28-Day Mortality in Pediatric Septic Shock: A Prospective Cohort Study

**DOI:** 10.7759/cureus.68912

**Published:** 2024-09-07

**Authors:** Duy-Truong Khac Le, Phuong Minh Nguyen, Ly Cong Tran, Viet Trieu Nguyen

**Affiliations:** 1 Department of Pediatrics, Can Tho University of Medicine and Pharmacy, Can Tho City, VNM; 2 Department of Otolaryngology, Can Tho University of Medicine and Pharmacy, Can Tho City, VNM

**Keywords:** critical care, septic shock, mortality, lactate/albumin ratio, pediatrics

## Abstract

Background

Septic shock remains a leading cause of mortality in children. The lactate/albumin ratio (LAR) has emerged as a potential prognostic marker for mortality in septic shock, yet most existing research focuses on adults, with limited data available for pediatric populations, particularly in Vietnam.

Objectives

This study aims to evaluate the prognostic utility of the LAR in predicting 28-day mortality among children aged two months to 15 years with septic shock in Vietnam.

Methods

We conducted a prospective cohort study involving children diagnosed with septic shock at the largest pediatric intensive care unit (PICU) in the Mekong Delta, Vietnam, from July 2022 to June 2024. Clinical and laboratory parameters, including lactate and albumin levels, were measured at the time of septic shock diagnosis. Patients were followed for 28 days, with outcomes categorized as either survival or mortality. The prognostic performance of LAR was assessed through its discrimination and calibration capabilities.

Results

The 28-day mortality rate was 63.4%. LAR was significantly higher in non-survivors compared to survivors (p < 0.001). The area under the receiver operating characteristic curve (AUROC) for LAR was 0.91, indicating superior discriminatory power compared to lactate alone and comparable to albumin. Using a Youden index-derived cut-off of 1.84, LAR demonstrated a sensitivity of 84.6% and a specificity of 80%. Kaplan-Meier analysis and log-rank testing revealed significantly lower survival probabilities in children with LAR ≥1.84 (p < 0.05). The Hosmer-Lemeshow test confirmed good calibration of LAR in mortality prediction (p > 0.05).

Conclusion

The lactate/albumin ratio exhibits excellent discriminatory and calibration properties, making it a valuable tool for predicting 28-day mortality in pediatric septic shock. This ratio should be considered for routine use in clinical practice to improve prognostic assessments in this vulnerable population.

## Introduction

Septic shock is a leading cause of mortality in children, with death rates exceeding 80% in certain regions [1]. The acute and life-threatening nature of septic shock demands that clinicians employ accurate and timely methods to predict patient outcomes. Various mortality prediction scoring systems have been developed for pediatric patients [2]. However, prognosticating outcomes in critically ill children with unstable hemodynamics remains a formidable challenge. Existing models often rely on complex physiological variables, which can be difficult to collect in critically ill patients [2,3]. Moreover, the limitations of hospital resources may prevent the full implementation of these intricate scoring systems.

In light of these challenges, there is a clear need for simpler, less invasive methods that offer acceptable accuracy in predicting outcomes for children with septic shock. The lactate/albumin ratio (LAR) has been highlighted in numerous studies as a promising predictor of mortality [4-7]. Despite this, the majority of research on LAR has focused on adult populations, with scant attention given to pediatric septic shock. Additionally, there is a significant lack of data on the utility of LAR in Vietnam, particularly among children suffering from septic shock.

Given the simplicity and practicality of LAR in clinical settings, we hypothesize that it holds significant prognostic value for mortality in pediatric septic shock. Therefore, this study was undertaken to evaluate the prognostic ability of the LAR in predicting 28-day mortality in children with septic shock.

## Materials and methods

Study design and subjects

This prospective cohort study was conducted at the Pediatric Intensive Care Unit (PICU) of Can Tho Children's Hospital, the largest pediatric facility in the Mekong Delta, from July 2022 to June 2024. The study population comprised children aged two months to 15 years who were diagnosed with septic shock according to the criteria set forth by the International Pediatric Sepsis Consensus Conference (IPCSS) 2005 [8].

Exclusion criteria included: (1) children diagnosed at another hospital and admitted in stable condition; (2) cases lacking complete data, particularly blood lactate and albumin levels, within six hours of diagnosis; and (3) children who died within six hours of diagnosis.

Sepsis was diagnosed based on the presence of two or more systemic inflammatory response syndrome (SIRS) criteria, alongside clinical and laboratory evidence suggestive or confirmatory of infection [8]. Septic shock was defined as sepsis accompanied by cardiovascular dysfunction [8].

The sample size was calculated using the formula for assessing the accuracy of a single quantitative diagnostic test [9], based on an AUC of 0.78 for the LAR from a previous meta-analysis [10]. A non-survivor-to-survivor ratio of 3:2 was estimated from mortality rates reported in a recent study conducted at our institution [2]. The significance level (α) was set at 0.05, with a power (1-β) of 90% and an estimated attrition rate of 5%. This yielded a required sample size of 41 cases. A non-probability sampling approach was employed, and all eligible patients meeting the inclusion criteria were included over the study period.

Data collection

Clinical data were collected at the time of septic shock diagnosis, including patient demographics (age, sex) and vital signs, such as temperature, heart rate, respiratory rate, blood pressure, capillary refill time, and Glasgow Coma Scale scores.

Laboratory data were obtained within six hours of septic shock diagnosis. For parameters measured multiple times during this period, the most clinically significant (worst) value was selected for analysis. Venous blood samples were used for biochemical assessments. Blood lactate levels were measured using an enzymatic photoflourometry method on an AU480 Chemistry Analyzer (Beckman Coulter, Inc., Brea, CA, United States), while serum albumin concentrations were determined using the Bromocresol Green (BCG) colorimetric method on the same analyzer.

Patients were monitored from the time of septic shock diagnosis until the outcome, either mortality or survival, was determined, with a maximum follow-up duration of 28 days. The follow-up duration was calculated from the time of diagnosis to the determination of the primary endpoint. Mortality was defined as death occurring during hospitalization or within 24 hours of discharge. Data collection and management were performed using Epidata 3.1 software (EpiData Association, Odense, Denmark).

Statistical analysis

Data were analyzed using R software version 4.4.1 (R Foundation for Statistical Computing, Vienna, Austria), with results presented in tables and graphs. Categorical variables are expressed as frequencies and percentages. Quantitative variables are presented as mean (standard deviation, SD) for normally distributed data, or as median (interquartile range, IQR) for non-normally distributed data. Differences between categorical variables were analyzed using the Chi-square or Fisher's exact test, while comparisons of quantitative variables were made using the independent-sample T-test or Wilcoxon rank-sum (Mann-Whitney) test, depending on the distribution of the data. Statistical significance was defined as p < 0.05.

The prognostic ability of the LAR, lactate, and albumin for mortality was evaluated through two key aspects: discrimination and calibration. Discrimination was assessed using the receiver operating characteristic (ROC) curve, comparing the area under the ROC curve (AUROC) for lactate, albumin, and LAR. Differences in AUROC between pairs of indicators were evaluated using the DeLong and Bootstrap tests, with the Bootstrap method involving 1,000 replications. The Youden index was utilized to determine optimal cut-off values for lactate, albumin, and LAR, maximizing sensitivity and specificity. Based on these cut-off values, we calculated the positive predictive value (PPV), negative predictive value (NPV), sensitivity, specificity, positive likelihood ratio (PLR), negative likelihood ratio (NLR), and overall accuracy for each indicator. Using the identified cut-off value for LAR, the sample was divided into two groups, and Kaplan-Meier curves along with the Log-Rank test were used to compare survival probabilities over time between the groups. Median survival time was also determined. Calibration of the lactate, albumin, and LAR indicators was assessed using the Hosmer-Lemeshow test, with the χ² value, degrees of freedom, p-value, and percentage accuracy recorded. A p-value > 0.05 indicated no significant difference between observed and predicted values, suggesting a good fit of the model.

Ethics statement

This study was approved by the Ethics Committee in Biomedical Research of Can Tho University of Medicine and Pharmacy (No. 22.158.HV/PCT-HĐĐĐ) on July 29, 2022. Prior to the commencement of the study, informed consent was obtained from the parents or legal guardians of all participants. They were assured of their right to withdraw their child from the study at any time without affecting the child's medical care. All data concerning the participating children were kept confidential.

## Results

General characteristics

A total of 41 patients met the inclusion criteria and were enrolled in the study. During the 28-day follow-up period, the overall mortality rate was 63.4% (26/41). The median age of the cohort was 21 months (IQR: 7-102), with 39% (16/41) of the patients being infants (<12 months). There were no statistically significant differences in age or sex distribution between the survival and non-survival groups. Underlying diseases were present in four patients (9.8%), with an equal distribution between the survival and non-survival groups (two patients per group), and no significant difference was detected (Fisher’s exact test, p=0.615). The duration of follow-up was significantly shorter in the non-survival group compared to the survival group (p < 0.001; Table 1).

**Table 1:** General Characteristics of Participants. Data are presented as n (%), mean ± SD, or median (IQR), as indicated for each variable; *p-value < 0.05 was considered significant; ^a^Wilcoxon test, with W value; ^b^Chi-Square test for independence, with ꭓ2-statistic (df) value; ^c^Fisher’s Exact test; PaO_2_/FiO_2_: The ratio of partial pressure of oxygen in arterial blood to the fraction of inspiratory oxygen concentration; SD: standard deviation; IQR: interquartile range.

Characteristics		All patients (n=41)	Survivors (n=15)	Non-survivors (n=26)	Statistical Value	p-value
Age (months), median (IQR)		21 (7-102)	20 (7.5-87)	27 (7-108)	180.5	0.704^a^
Male sex, n (%)		24 (58.5)	9 (60)	15 (57.7)	1.26e-31 (1)	0.999^b^
Temperature (⁰C), median (IQR)		40 (39-40)	40 (39-40)	40 (39-40)	209	0.699^a^
Heart rate (beats/minute), median (IQR)		185 (160-200)	185 (170-200)	184 (160-200)	203	0.838^a^
Systolic blood pressure (mmHg), median (IQR)		60 (0-80)	70 (25-80)	58 (0-80)	240.5	0.204^a^
Mean blood pressure (mmHg), median (IQR)		46.7 (0-66.7)	56.7 (18.3-66.7)	46.7 (0-66.7)	237.5	0.24^a^
Respiratory rate (breaths/minute), median (IQR)		46 (32-52)	47 (33.5-52)	46 (32-51.5)	210	0.694^a^
Capillary refill time (second), median (IQR)		4 (4-5)	4 (3-4)	4 (4-5)	10	<0.001^a,*^
Glasgow coma scale, median (IQR)		9 (8-11)	12 (10-12)	8 (8-9)	352.5	<0.001^a,*^
White blood cell (x10^3^/mm^3^), mean ± SD		14.4 ± 7.6	12.3 ± 7.8	15.6 ± 7.5	136	0.19^a^
Platelet < 150x10^3^/mm^3^, n (%)		16 (39.1)	1 (6.7)	15 (57.7)	_	0.001^c,*^
Procalcitonin (ng/mL), median (IQR)		64.5 (2.95-100)	5.5 (1.3-100)	56.2 (11.7-100)	124.5	0.257^a^
Blood culture positive, n (%)		7 (17.1)	4 (26.7)	3 (11.5)	_	0.390^c^
PaO_2_/FiO_2_, mean ± SD		288 ± 162.2	351.3 ± 160.2	251.4 ± 154.7	267	0.052^a^
Site of infection, n (%)	Gastrointestinal	23 (56.1)	8 (53.3)	15 (57.7)	_	0.914^c^
	Respiratory	8 (19.5)	4 (26.7)	4 (15.4)		
	Central nervous system	3 (7.3)	1 (6.7)	2 (7.7)		
	Unknown	7 (17.1)	2 (13.3)	5 (19.2)		
Length of observation (days)		4.3 (1.2-14.3)	18.8 (14.3-24.7)	1.5 (0.9-4.1)	385	<0.001^a,*^

At the time of diagnosis, patients exhibited elevated temperatures, tachycardia, and hypotension, with no significant differences between the survival and non-survival groups. However, the non-survival group had a significantly prolonged capillary refill time (CRT) and lower Glasgow Coma Scale (GCS) scores compared to the survival group. In terms of laboratory findings, thrombocytopenia (platelet count <150×10³/mm³) was more prevalent in the non-survival group. The overall rate of positive blood cultures was low, with no significant difference between the two groups. The primary source of infection was the gastrointestinal tract, followed by the respiratory tract (Table 1).

Discrimination of lactate, albumin, and LAR in predicting 28-day mortality

Regarding the assessment of discrimination at the time of diagnosis, we observed elevated blood lactate levels in patients, with a median value of 6.6 mmol/L (IQR: 4.2-8.7), accompanied by decreased blood albumin levels, with a median value of 2.8 g/dL (IQR: 2.1-3.3). This combination resulted in an elevated LAR, with a median value of 2.24 (IQR: 1.6-3.7) (Table 2).

**Table 2:** Comparison of Lactate, Albumin, and LAR Between Survival and Non-Survival Groups. ^a^Wilcoxon test with W value; ^*^p-value < 0.05 was considered significant; LAR: lactate/albumin ratio; IQR: interquartile range.

	Total (n=41)	Survivors (n=15)	Non-survivors (n=26)	Statistical value	p-value
Lactate (mmol/L), median (IQR)	6.6 (4.2-8.7)	3.3 (1.9-5.8)	7.7 (6.4-10.7)	60	<0.001^a,*^
Albumin (g/dL), median (IQR)	2.8 (2.1-3.3)	3.4 (3-3.6)	2.4 (2-2.7)	363	<0.001^a,*^
LAR, median (IQR)	2.24 (1.6-3.7)	0.92 (0.6-1.7)	3.7 (2.1-5.1)	36	<0.001^a,*^

**Figure 1:**
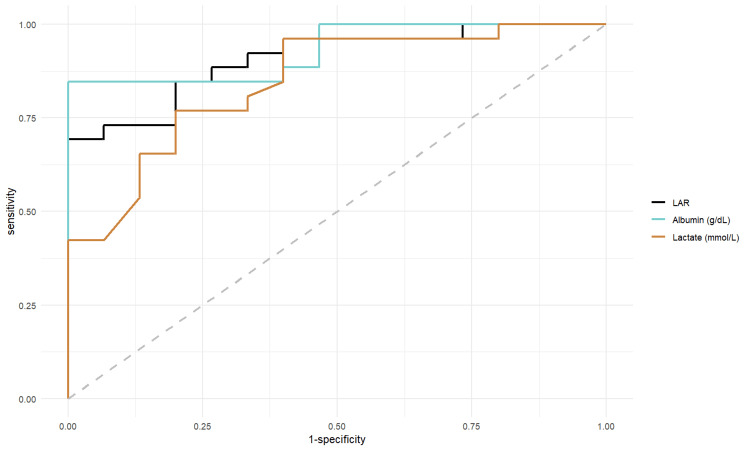
AUROC Analysis of LAR, Albumin, and Lactate. LAR: lactate/albumin ratio; AUROC: The area under the receiver operating characteristic curve.

The non-survival group exhibited significantly higher lactate and lactate/albumin ratio, as well as lower albumin levels, compared to the survival group (p < 0.001), as shown in Table 2. Analysis of the AUROC for these three indicators in predicting 28-day mortality revealed that all three ROC curves were significantly distinct from the reference line (Table 3, Figure 1).

**Table 3:** Area Under the Curve Analysis of Lactate, Albumin, and LAR. ^*^p-value < 0.05 was considered significant; LAR: lactate/albumin ratio; AUROC: The area under the receiver operating characteristic curve; CI: confidence intervals.

	AUROC	95% CI	p-value
LAR	0.91	0.82-0.99	<0.001^*^
Lactate	0.85	0.73-0.97	<0.001^*^
Albumin	0.93	0.86-1.00	<0.001^*^

The AUROC for the lactate/albumin ratio was significantly greater than that for lactate alone, as confirmed by DeLong and Bootstrap tests. However, there was no significant difference between the AUROC of albumin and that of the other two indicators (Table 4). 

**Table 4:** Pairwise Comparison of AUROC Curves for Predicting Mortality Using Lactate, Albumin, and LAR. ^*^p-value < 0.05 was considered significant; CI: confidence intervals; LAR: lactate/albumin ratio; AUROC: The area under the receiver operating characteristic curve.

	AUROC Difference	DeLong Test			Bootstrap Method	
		Z	95% CI	p-value	95% CI	p-value
LAR and lactate	0.062	1.98	0.001-0.12	0.048^*^	0.02-0.16	0.014^*^
LAR and albumin	-0.023	-0.428	-0.13-0.08	0.669	-0.15-0.08	0.65
Lactate and albumin	-0.085	-1.131	-0.23-0.06	0.258	-0.26-0.05	0.25

As shown in Table 5, the LAR demonstrates higher sensitivity, predictive value, likelihood ratio, and accuracy compared to lactate alone. The accuracy of lactate was also lower than that of the others.

**Table 5:** Discriminatory Ability of Lactate, Albumin, and LAR in Predicting 28-Day Mortality Se: sensitivity; Sp: specificity; PPV: positive predictive value; NPV: negative predictive value; PLR: positive likelihood ratio; NLR: negative likelihood ratio; ACC: accuracy; CI: confidence intervals; LAR: lactate/albumin ratio.

Parameters	Lactate	Albumin	LAR
Optimal cut-off	6.1	2.87	1.84
Se (%),(95% CI)	76.9 (56.4-91.0)	84.6 (65.1-95.6)	84.6 (65.1-95.6)
Sp (%), (95% CI)	80 (51.9-95.7)	100 (78.2-100)	80 (51.9-95.7)
PPV (%), (95% CI)	98.7 (96.3-99.5)	100 (84.6-100)	98.8 (96.7-99.6)
NPV (%), (95% CI)	15.4 (8.0-27.8)	25.5 (12.2-45.7)	21.5 (9.7-41.1)
PLR (95% CI)	3.9 (1.4-10.8)	-	4.2 (1.5-11.8)
NLR (95% CI)	0.3 (0.1-0.6)	0.2 (0.1-0.4)	0.2 (0.1-0.5)
ACC (%), (95% CI)	77.1 (61.3-88.7)	85.4 (70.9-94.5)	84.4 (69.7-93.8)

**Figure 2:**
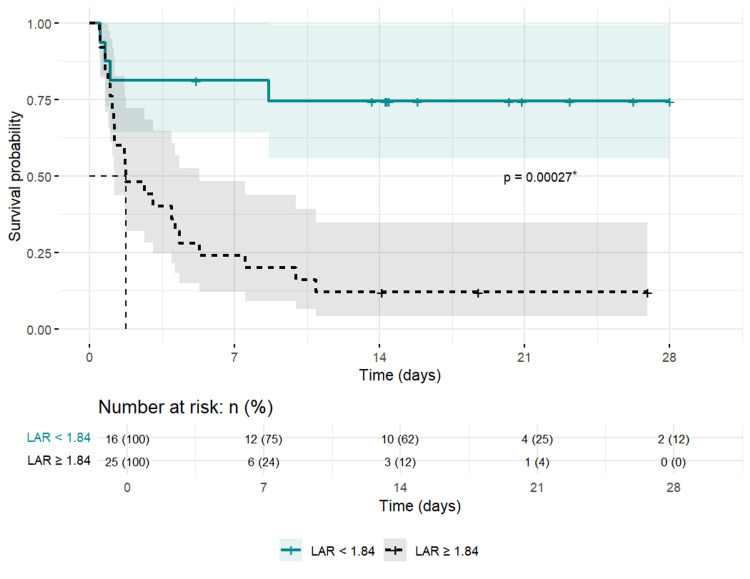
Kaplan-Meier Survival Curve and Risk Table Stratified by LAR for 28-Day Survival. ^*^Log-rank test; LAR: lactate/albumin ratio.

The group with a LAR ≥1.84 exhibited a significantly higher risk of mortality at all follow-up time points (p < 0.05). Moreover, the median survival time for this group was 1.75 days (Figure 2).

Calibration of lactate, albumin, and LAR in predicting 28-day mortality

The Hosmer-Lemeshow Goodness-of-Fit test indicated no significant differences between observed and predicted mortality values for lactate, albumin, and LAR (all p-values > 0.05). However, for lactate, there was a larger discrepancy between observed and predicted cases within the probability range of 0.303 to 0.465 compared to other ranges. Similarly, for albumin, the probability range of 0.192 to 0.562 showed a notably larger difference in the mortality group between observed and predicted cases. Despite these discrepancies, all three indicators, including lactate, albumin, and LAR, demonstrated good overall calibration, with accuracy rates of 75.6%, 75.6%, and 80.5%, respectively (Table 6).

**Table 6:** Hosmer-Lemeshow Test for Lactate, Albumin, and LAR in Predicting 28-Day Mortality ^*^p-value ≥ 0.05 was considered good fit; O: Observed; E: Expected; LAR: lactate/albumin ratio.

Lactate			Albumin			LAR		
Probability interval	O	E	Probability interval	O	E	Probability interval	O	E
[0.119-0.613]	1	0.69	[0.001 – 0.106]	0	0.29	[0.057-0.113]	1	0.40
(0.613-0.303]	0	0.86	(0.106-0.192]	1	0.58	(0.113-0.169]	0	0.54
(0.303-0.465]	3	1.68	(0.192-0.423]	3	1.31	(0.169-0.426]	2	1.49
(0.465-0.597]	2	2.24	(0.423-0.562]	0	2.21	(0.426-0.54]	2	1.97
(0.597-0.716]	3	2.74	(0.562-0.753]	2	2.75	(0.54-0.695]	2	2.59
(0.716-0.811]	3	3.07	(0.753-0.848]	4	3.23	(0.695-0.914]	3	3.29
(0.811-0.819]	3	3.28	(0.848-0.962]	4	3.76	(0.914-0.971]	4	3.79
(0.819-0.945]	3	3.60	(0.962-0.985]	4	3.91	(0.971-0.995]	4	3.93
(0.945-0.987]	4	3.86	(0.985-0.996]	4	3.96	(0.995-0.999]	4	3.99
(0.987-1]	4	3.98	(0.996-1]	4	3.99	(0.999-1]	4	3.99
χ² = 4.51; df = 8; p = 0.81^*^; Overall percentage: 75.6%			χ² = 10.8; df = 8; p = 0.21^*^; Overall percentage: 75.6%			χ² = 2.71; df = 8; p = 0.95^*^; Overall percentage: 80.5%		

## Discussion

General characteristics

The median age of our cohort was 21 months, with a predominance of male patients. These findings are consistent with previous studies by Nguyen et al. and Evren et al., which also reported similar age and gender distributions in pediatric septic shock populations [11,12]. The observed demographic trends may be attributed to genetic, endocrine, and immunological differences in younger patients, which could influence susceptibility and outcomes in septic shock [13,14].

The positive blood culture rate in our study was relatively low at 17.1%, with no significant difference observed between the survival and non-survival groups. This finding aligns with the study by Hazwani et al., which reported a positive blood culture rate of 14.3% and found a higher mortality rate among patients with positive cultures (p = 0.004) [15]. Conversely, Kim et al. reported no significant differences in clinical outcomes or mortality between patients with positive and negative blood cultures [16]. The low positive culture rate in our study could be attributed to several factors, including the administration of antibiotics prior to blood culture collection, which can reduce the likelihood of detecting causative pathogens. Additionally, some children in our study succumbed to septic shock before blood culture results were available, leading to the cancellation of cultures before the required incubation period was completed. These factors, while reflective of real-world clinical practices, may have influenced our findings.

In terms of infection sources, the digestive tract was the most common origin in our study, accounting for 56.1% of cases, followed by the respiratory tract at 19.5%. The source of infection remained unidentified in 17.1% of cases, with no significant difference between survival and non-survival groups. These findings are consistent with those of Nguyen et al. in Vietnam [11]. However, Uppala et al. reported equal proportions of digestive and respiratory tract infections (25% each) and a lower rate of unidentified sources (14.3%) [17]. These differences may be attributed to variations in disease patterns and healthcare practices across different geographic regions.

The mortality rate observed in our study was notably high at 63.4%. This is comparable to the 50.8% mortality rate reported by Wolfler et al. in Italy [18], but lower than the 88.2% mortality rate recorded by Rusmawatiningtyas et al. in Indonesia [1]. The variation in mortality rates across studies highlights the influence of local disease patterns, healthcare infrastructure, and the timing of interventions on patient outcomes.

Our data also revealed that the non-survival group had a significantly shorter follow-up period compared to the survival group (p < 0.001). Most deaths occurred within the first seven days of follow-up, underscoring the rapid progression of septic shock in critically ill children. Early mortality in these cases likely reflects the severe and often irreversible nature of the disease, leading to shorter hospital stays. Conversely, the extended hospital stays in the survival group were often due to the need for prolonged resuscitation, management of complications, and addressing sequelae. These findings illustrate the substantial healthcare burden imposed by septic shock on both survivors and non-survivors.

Discrimination of three indices for predicting 28-day mortality

Discrimination of Lactate

In our study, elevated blood lactate levels were observed in patients with septic shock, with significantly higher levels recorded in the non-survivor group (7.7 mmol/L) compared to the survivor group (3.3 mmol/L) (p < 0.001). These lactate levels are notably higher than those reported in other studies. For instance, Kim et al. documented an average lactate level of 3.92 ± 3.67 mmol/L at the time of shock in 65 children, with significantly higher levels in the non-survivor group (6.16 ± 4.87 mmol/L) compared to the survivor group (3.13 ± 2.79 mmol/L) (p = 0.015) [19]. In contrast, Abdelaziz et al. reported a median lactate level of 5.2 mmol/L (IQR: 2.7-18), with no significant difference between outcome groups (p = 0.59) [20]. The discrepancy between our findings and those of Abdelaziz et al. may be attributed to differences in the study populations; our study focused exclusively on children with septic shock, whereas Abdelaziz's study included children with severe sepsis [20]. Additionally, a study by Al-Eyadhy et al. involving 47 pediatric patients noted that the percentage of children with initial blood lactate levels higher than 5 mmol/L was significantly higher in the non-survivor group compared to the survivor group (p = 0.02) [21]. This variation in lactate levels across studies suggests that, while lactate levels at the time of shock are influenced by several factors, they remain a valuable marker for differentiating and predicting mortality in pediatric septic shock.

Our analysis demonstrated that the AUROC for lactate was 0.85 (95% CI: 0.73-0.97, p < 0.001), indicating a strong predictive ability for mortality. Using the Youden index, we identified an optimal cut-off point of 6.1 mmol/L. At this threshold, lactate predicted mortality with a sensitivity of 76.9%, specificity of 80%, positive predictive value of 98.7%, and negative predictive value of 15.4%. These findings are slightly higher than those reported by Cakir et al., who found an AUROC of 0.816 (95% CI: 0.792-0.838, p < 0.001) with a cut-off point of 2.2 mmol/L, and corresponding sensitivity, specificity, positive predictive value, and negative predictive value of 78%, 77%, 72%, and 83%, respectively [5]. The differences in results can likely be attributed to Cakir et al.'s focus on adult sepsis, where physiological differences and the severity of the condition may have influenced the outcomes [5].

Discrimination of Albumin

Our study observed that albumin levels were significantly lower in the non-survival group compared to the survival group. This finding is consistent with results from other studies across different age groups, such as those by Bou Chebl et al. [4] and Cakir et al. [5]. Collectively, our research and that of others have consistently shown a marked decrease in blood albumin levels in patients who do not survive, suggesting that hypoalbuminemia is a critical factor in predicting mortality in pediatric septic shock. Given that albumin levels at the time of diagnosis can vary significantly depending on age and regional disease patterns, assessing the degree of hypoalbuminemia is crucial for mortality prediction in these patients.

In our study, the AUROC for albumin was 0.93 (95% CI: 0.86-1.00, p < 0.001), indicating a strong predictive capability for mortality. Using the Youden index, we identified an optimal cut-off point of 2.87 g/dL. At this threshold, albumin predicted mortality with a sensitivity of 84.6%, specificity of 100%, positive predictive value of 100%, and negative predictive value of 25.5%. In comparison, Cakir et al. reported an AUROC of 0.812 (95% CI: 0.788-0.834, p < 0.001) for albumin, with a cut-off point of 2.6 g/dL, and corresponding sensitivity, specificity, positive predictive value, and negative predictive value of 72%, 78%, 71%, and 79%, respectively [5]. The differences in findings may be attributed to variations in study populations and disease severity, as Cakir’s study focused on adult patients with sepsis.

Discrimination of the Lactate/Albumin Ratio

Our study found that the LAR was significantly higher in the non-survival group compared to the survival group, with values exceeding those reported in other studies. For instance, Shadvar et al. reported a LAR of 0.259 ± 0.053 in the non-survival group, which was higher than the survival group's 0.149 ± 0.036 (p < 0.001) in a study of 151 patients with septic shock [22]. Similarly, Cakir et al., in a study of 1,136 sepsis patients, found LAR values of 1.27 (0.35-5.82) in the non-survival group and 0.44 (0.1-2.45) in the survival group, with a significant difference between the groups (p < 0.001) [5]. However, Iskandar et al. reported no significant difference in LAR between the non-survival (1.35 ± 0.7) and survival (1.01 ± 0.59) groups (p = 0.119) in a study of 58 sepsis patients [23]. These variations could be due to differences in patient age and disease severity across studies.

The complex interplay of factors influencing lactate production and metabolism in children with septic shock, including tissue hypoxia, anaerobic metabolism, decreased lactate clearance, and microcirculatory dysfunction, contributes to elevated lactate levels. During septic shock, lactate is released from various sources and transported across different tissues, affecting its overall concentration [20,24,25]. Meanwhile, hypoalbuminemia in septic shock is attributed to vasodilation, vascular leakage, liver dysfunction, and fluid resuscitation, all of which are associated with poor prognosis [26]. However, albumin levels can also be influenced by the patient's nutritional status and chronic inflammation, which limits its utility as an independent prognostic marker [27]. The elevated LAR in our study suggests that the children had severe infections, prolonged tissue hypoxia, and metabolic disturbances at multiple levels, possibly contributing to the higher mortality rate observed.

In our study, the AUROC for LAR was 0.91 (95% CI: 0.82-0.99, p < 0.001), indicating an excellent ability to predict mortality. The optimal cut-off point, determined by the Youden index, was 1.84. At this threshold, the LAR predicted mortality with a sensitivity of 84.6%, specificity of 80%, positive predictive value of 98.8%, and negative predictive value of 21.5%. In contrast, a meta-analysis by Yoon et al., which synthesized data from eight studies, reported a pooled sensitivity of 0.71 (95% CI: 0.54-0.84) and specificity of 0.68 (95% CI: 0.58-0.76) [10]. The wide range of optimal LAR thresholds reported in different studies (0.115 to 1.735) likely reflects variations in test types, sample collection methods, patient characteristics, and clinical contexts [10].

When comparing AUROC values using the DeLong and Bootstrap methods, our study found that the AUROC of LAR was significantly greater than that of lactate alone, with both methods confirming statistical significance. However, there was no significant difference in AUROC between albumin and the other two indices. The Bootstrap method, which involves resampling with replacement, does not require normal distribution assumptions and is well-suited for small sample sizes, as in our study. This flexibility makes it an appropriate method for our analysis, leading to the conclusion that the AUROC of LAR is superior to lactate and comparable to albumin.

The LAR demonstrated higher sensitivity than lactate and was equivalent to albumin at the identified cut-off points. While the specificity of LAR was similar to that of lactate, it was lower than that of albumin. The positive likelihood ratio of LAR also exceeded that of lactate. Cakir et al. similarly reported that the AUROC of LAR was greater than that of lactate and albumin [5]. However, Bou Chebl's study noted that in patients with sepsis, the AUROC of LAR was greater than that of lactate but smaller than that of albumin, and that all three indices had ROC curves close to the reference line when examining septic shock patients separately (p > 0.05) [4]. These discrepancies across studies could be due to differences in testing timing, severity, and the age of study participants.

The Kaplan-Meier curve (Figure 2) further illustrates the cumulative survival probability over time between groups stratified by the LAR threshold. The group with a LAR below 1.84 had a significantly higher survival probability over the 28-day follow-up period compared to the group with a LAR ≥1.84. The log-rank test (p < 0.001) confirmed a statistically significant difference in survival probabilities between these groups.

Overall, our findings suggest that LAR is a more effective discriminator for predicting 28-day mortality than lactate and is comparable to albumin in children with septic shock at the time of diagnosis.

Calibration

The Hosmer-Lemeshow Goodness-of-Fit test revealed no significant differences between the observed and predicted values for all three indices, with p-values > 0.05. This suggests that the data are well-suited for lactate, albumin, and LAR calibration. Nguyen et al. also reported that lactate demonstrated good calibration ability for mortality prediction, with a Hosmer-Lemeshow test result of χ² = 12.442, df = 8, p = 0.133. Furthermore, their study indicated that lactate maintained good calibration across different subgroups, such as age, gender, and primary diagnosis (sepsis/non-sepsis), with p-values > 0.05 [28].

We recorded χ² values for LAR, lactate, and albumin at 2.71, 4.51, and 10.8, respectively, with overall accuracy rates of 80.5%, 75.6%, and 75.6%. Among the three indices, LAR had the lowest χ² value, indicating the smallest difference between observed and expected values, while albumin had the highest χ² value. The smaller χ² value for LAR suggests a better fit with the data, reflecting its superior goodness-of-fit and accuracy compared to lactate and albumin. Therefore, while all three indices exhibit good calibration, LAR demonstrates the highest calibration ability.

In summary, our study shows that all three indices possess strong discriminatory power, with LAR providing better discrimination between survival and non-survival groups than lactate and performing on par with albumin. When evaluating calibration ability, LAR also surpasses the other two indices in accuracy for predicting 28-day mortality. LAR combines the robust discriminatory capacity of albumin with the superior calibration ability of lactate, making it an excellent tool for predicting 28-day mortality in children with septic shock and applicable in clinical settings.

Strengths and limitations

This study takes a comprehensive and methodical approach by comparing the LAR with traditional biomarkers, such as lactate and albumin, and employing robust statistical analyses, including ROC curves and the Hosmer-Lemeshow test, to thoroughly assess predictive performance. The use of the Bootstrap method further enhances the reliability and validity of the findings, even with a limited sample size, offering valuable insights that contribute to the existing literature on prognostic indicators in pediatric septic shock. However, this study has limitations, including a relatively small sample size and a single-center design, which may affect the generalizability of the results. To strengthen these findings, future research should include larger sample sizes and multi-center studies. Additionally, variations in the timing of blood collection due to resuscitation efforts during shock may have introduced confounding factors that influenced the outcomes. Due to limited resources in our setting and the urgency of resuscitation at the time of septic shock diagnosis, we were unable to collect comprehensive data on the causes of septic shock. Future studies should address this gap to provide a more complete perspective.

## Conclusions

Our study indicates that the LAR demonstrates excellent discriminatory ability and good calibration for predicting 28-day mortality in children with septic shock. However, further research with larger sample sizes and multi-center designs is recommended to validate these findings and enhance their generalizability.
